# The Molecular Mechanisms of HLA-G Regulatory Function on Immune Cells during Early Pregnancy

**DOI:** 10.3390/biom13081213

**Published:** 2023-08-03

**Authors:** Jia Mao, Ying Feng, Xiaofeng Zhu, Fang Ma

**Affiliations:** 1 Center for Translational Medicine, Key Laboratory of Birth Defects and Related Diseases of Women and Children (Sichuan University), Ministry of Education, West China Second University Hospital, Sichuan University, Chengdu 610041, China; maojia@stu.scu.edu.cn; 2 Key Laboratory of Bio-Resource and Eco-Environment of Ministry of Education, State Key Laboratory of Biotherapy and Cancer Center, College of Life Sciences, Sichuan University, Chengdu 610064, China; 3 Department of Histology, Embryology and Neurobiology, West China School of Basic Medical Sciences & Forensic Medicine, Sichuan University, Chengdu 610041, China; yingfeng27@scu.edu.cn; 4 Department of Obstetrics and Gynecology, West China Second University Hospital, Sichuan University, Chengdu 610041, China

**Keywords:** HLA-G, immune cell, NK cell, maternal-fetal immune interactions

## Abstract

Human leukocyte antigen-G (HLA-G) is a non-classical human major histocompatibility complex (MHC-I) molecule with the membrane-bound and soluble types. HLA-G is primarily expressed by extravillous cytotrophoblast cells located at the maternal–fetal interface during pregnancy and is essential in establishing immune tolerance. This review provides a comprehensive understanding of the multiple molecular mechanisms by which HLA-G regulates the immune function of NK cells. It highlights that HLA-G binds to microRNA to suppress NK cell cytotoxicity and stimulate the secretion of growth factors to support fetal growth. The interactions between HLA-G and NK cells also activate senescence signaling, promoting spiral artery remodeling and maintaining the balance of maternal–fetal immune responses. In addition, HLA-G can inhibit the function of decidual T cells, dendritic cells, and macrophages. Overall, the interaction between trophoblast cells and immune cells mediated by HLA-G plays a crucial role in understanding immune regulation at the maternal–fetal interface and offers insights into potential treatments for pregnancy-related diseases.

## 1. Introduction

Approximately four days after fertilization, the zygote undergoes the cleavage process to form a blastocyst. The blastocyst is composed of the inner cell mass and the outer trophoblast cell. The trophoblast cells close to the inner cell mass are responsible for attaching to the epithelium of the endometrium. After the trophoblast successfully adheres to the uterus, the blastocyst enters the invasion stage. The cytotrophoblast gradually forms villi, extending to the mother’s uterus through the outer syncytiotrophoblast and invading the uterine blood vessels and glands. When tertiary villi are formed, the cytotrophoblast at the tip of the villi differentiates to form invasive extravillous trophoblasts (EVTs) [1]. EVTs possess the ability to invade the spiral arteries of the myometrium and the interstitium of the uterus. The syncytiotrophoblast in the outer layer is for nutrient transport and gas exchange between the mother and the fetus. Moreover, it also secretes human chorionic gonadotrophin (hCG), progesterone, and placental lactogen to maintain pregnancy [2]. The cytotrophoblast and syncytiotrophoblast invade the maternal blood vessels within the endometrium, initiating the formation of the fetal portion of the placenta.

Along with the invasion of trophoblasts, the endometrium also undergoes dramatic changes, including the differentiation of endometrial stromal fibroblast cells (ESCs) into decidual stromal fibroblast cells (DSCs) with more nutrients, more angiogenesis, and an expansion of the uterine glands. The above process is called endometrium decidualization [3]. Initially, the transformation occurs around the implanting blastocyst and subsequently spreads throughout the entire endometrium. Eventually, the decidua beneath the chorion frondosum, referred to as decidua basalis, constitutes the maternal component of the placenta.

In the context of pregnancy, the embryo represents a unique semi-allograft [4] as it inherits half of its genes from the mother and the other half from the father. Remarkably, the maternal immune system does not reject the invading fetal EVTs during the endometrial invasion process. This highlights the critical importance of a balanced interaction between decidual immune cells and trophoblasts during implantation and throughout pregnancy. The molecular mechanisms underlying this interaction at the maternal–fetal interface have garnered significant interest in the field of implantation research.

HLA-G, a non-classical HLA class I molecule, was first detected in placental trophoblast cells and plays a crucial role in maternal–fetal immune tolerance [5]. The immunomodulatory function of HLA-G is divided into two aspects: direct binding to cell receptors on various immune cells and indirect regulation via HLA-G-dependent suppressor cells. Moreover, HLA-G interacts with NK cells, T cells, dendritic cells (DCs), and macrophage cells and may influence angiogenesis and cell migration. Therefore, HLA-G is essential for early placental development and successful pregnancy. This review article focuses on elucidating the mechanisms underlying the interaction between HLA-G and decidual NK cells, as well as other immune cells, while also summarizing the relevant signaling pathways involved in these processes.

## 2. The General Expression and Immune-Suppressive Function of HLA-G in Tumors and Autoimmune Diseases

### 2.1. The General Expression of HLA-G

HLA-G, the first identified HLA class I molecule on chorionic cytotrophoblast cell membranes [6], possesses a unique characteristic that results in different isoforms: the post-transcriptional alternative splicing of mRNA from a single HLA-G gene (Table 1). Since it was discovered in 1987, seven isoforms have been identified, including four membrane-bound proteins (HLA-G1 to HLA-G4) and three soluble proteins (HLA-G5 to HLA-G7). Under normal conditions, the expression of HLA-G is restricted, mainly in placental trophoblast cells [7]. Interestingly, the HLA-G protein has also been found in the male reproductive system and seminal plasma [8,9]. Furthermore, HLA-G expression has been observed in other immune-privileged sites, such as the thymus [10], cornea [11], and pancreatic islets [12]. While HLA-G expression is typically restricted to specific tissues, its expression can be induced under various pathological conditions, including cancer [13,14,15], transplantation [16,17], multiple sclerosis, inflammatory diseases, and viral infections [18]. Under physiological conditions, the expression of HLA-G can also be detected in immune cells, such as HLA-G^+^ regulatory T lymphocytes (Tregs) (originated from the thymus) and HLA-G^+^ NK cells (derived from peripheral blood CD34^+^ hematopoietic progenitors) [19,20,21]. The expression of HLA-G on immune cells increases in disease conditions such as infection, cancer, and transplantation. For instance, in HIV-infected patients, a significant proportion of monocytes and T lymphocytes express HLA-G, and the interaction between soluble HLA-G (sHLA-G) and its ligand is known to play a pivotal role in immune cell dysfunction [22,23]. Increased numbers of HLA-G^+^ T cells and NK cells were detected in the peripheral blood of breast cancer patients, which are conducive to tumor development [21,24]. Higher HLA-G expression levels were observed in CD4^+^ T lymphocytes and DCs from patients undergoing liver and kidney transplantation, suggesting a potential immunosuppressive role in these transplantation processes [25,26].

Most studies have highlighted the crucial role of HLA-G as an immunomodulator in pregnancy [40]. During pregnancy, sHLA-G (HLA-G5 and HLA-G6) in the maternal circulation is mainly produced and secreted by trophoblast cells [41]. However, regulatory T cells and antigen-presenting cells (such as monocytes and DCs) may also contribute to the production of sHLA-G [42,43]. Papuchova et al. compared HLA-G^+^ EVT from human first trimester and term placental tissues and found that the three isolated types of HLA-G^+^ EVT showed significant changes in phenotype, gene expression, response to pro-inflammatory signals, and the induction of regulatory T cells. These findings suggest the importance of HLA-G expression levels during pregnancy for placental development and our understanding of pregnancy-related diseases [5].

HLA-G expressed at the maternal–fetal interface seems essential for regulating pregnancy outcomes, while the presence of sHLA-G in maternal plasma also holds significance. In pregnant women, the level of sHLA-G in plasma is significantly higher compared to non-pregnant women, with an increase observed during the first three months of pregnancy, peaking in the third month [43]. The elevated sHLA-G levels during pregnancy may be attributed to the shedding of HLA-G1 and HLA-G5 from EVT cells [44]. Notably, sHLA-G1 and HLA-G5 can be detected in the supernatant of follicular fluid samples (FFs), and sHLA-G1 can be detected in the supernatant of fertilized embryos, indicating a potential relationship between the presence of sHLA-G and successful implantation [45]. However, in one study, the authors detected the protein levels of sHLA-G in FFs and found that sHLA-G levels in FFs were not associated with successful implantation. From these results, we can see that the concentration of HLA-G varies greatly in different samples, so whether HLA-G is related to successful implantation is also worth exploring [46].

### 2.2. The Immune-Suppressive Function of HLA-G in Tumors and Autoimmune Diseases

HLA-G expression is frequently observed in tumors as well as various autoimmune diseases, suggesting its potential involvement in the pathogenesis of these conditions. In the context of cancer, HLA-G has been shown to confer protection to cancer cells against the cytotoxic activity of natural killer cells and cytotoxic T lymphocytes, enabling tumor immune escape, promoting tumor proliferation, and correlating with reduced patient survival. In autoimmune diseases, HLA-G plays a role in immune system regulation, and its polymorphisms have been strongly associated with susceptibility to these conditions [47].

Paul et al. first reported that HLA-G expression was explicitly observed in melanoma lesions but not in adjacent non-tumor tissues [48]. Subsequently, HLA-G expression has been detected in various tumor tissues but is typically absent in surrounding healthy tissues. Particularly, immune cells infiltrating the tumor microenvironment can also exhibit HLA-G expression [49]. The direct immunosuppressive effect induced by HLA-G may occur by binding inhibitory receptors expressed by various immune cells (NK cells, T cells, DCs, and macrophages) [50]. The underlying mechanism of interaction between tumor cells expressing HLA-G and immune cells is similar to that observed in trophoblast cells expressing HLA-G, as discussed in Part 2. For further insights into the regulatory mechanisms of HLA-G in tumor immune evasion, please refer to the comprehensive review by Liu [51].

Autoimmune diseases (AIDs) occur when the body’s immune system loses its ability to distinguish between self and non-self, leading to an immune response against its own tissues and cells. This immune dysregulation results in cellular destruction, tissue damage, and the manifestation of clinical symptoms [52]. HLA-G participates in immune regulation in various AIDs, including neurological diseases, systemic lupus erythematosus, and rheumatic diseases. In the case of multiple sclerosis (MS), an autoimmune disease that targets the central nervous system and leads to demyelination, there is mounting evidence of the high expression of HLA-G in immune cells found in the cerebrospinal fluid of MS patients [53]. This elevated HLA-G expression may have a beneficial effect in suppressing immune cell function. Additionally, sHLA-G, which is prominently present in the cerebrospinal fluid, may act as an anti-inflammatory molecule and, together with IL-10, play a role in regulating disease activity in MS [54,55]. For a comprehensive exploration of the role of HLA-G in other autoimmune diseases, please refer to the review by Contini [56].

Numerous studies have investigated the expression of HLA-G in both cancer and autoimmune diseases, highlighting its clinical significance. HLA-G expression has been closely associated with tumor progression and patient prognosis, making it a promising target for CAR-T cell therapy [57]. While there is currently no evidence supporting the use of sHLA-G levels in serum as biomarkers for MS, sHLA-G levels in cerebrospinal fluid have shown promise as prognostic markers for the disease. Although the role of HLA-G in tumor immune escape and autoimmune diseases has been extensively explored, it is particularly relevant to pregnancy, where it plays a crucial role in immune tolerance.

## 3. HLA-G and Immune Cells at the Maternal–Fetal Interface

### 3.1. Immune Cells at the Maternal–Fetal Interface

During the establishment of the maternal–fetal interface, the process of decidualization is accompanied by the infiltration of various immune cells, including uterine natural killer cells (uNKs), macrophages, and T cells [58]. The population of white blood cells in the endometrium undergoes variations throughout the menstrual cycle. The lowest numbers of white blood cells are typically observed during the proliferative phase of the endometrium, while the highest numbers are seen during the secretory phase.

During the late secretory stage and early pregnancy, the population of uterine natural killer cells (uNKs) undergoes a rapid increase, comprising approximately 70% of the leukocytes in the uterus. This population of uNKs reaches its peak in early pregnancy and subsequently decreases as the pregnancy progresses towards term [59,60]. The primary phenotype of uNK is characterized by CD56^bright^CD16^−^, which is different from the dominant CD56^dim^CD16^+^ NK cells in peripheral blood [61,62,63]. uNKs are generally considered hypotoxic as they do not express CD16 (a cytotoxicity marker) and produce minimal cytokines [64]. Apart from uNKs, the decidua also contains a small number of other immune cells, including macrophages (~20%), T cells (~10%), and DCs (1–2%) [65]. CD8^+^ T cells constitute the majority of T cells within the decidua. The cytolytic activity of CD8^+^ T cells is maintained during the proliferative phase but decreases during the secretory phase, although the overall number of CD8^+^ T cells remains relatively constant. Additionally, the number of immunosuppressive CD4^+^ Tregs in the decidua and peripheral blood increases from the first trimester onwards, contributing to an immunosuppressive environment [66,67].

The population of macrophages and DCs increases during the secretory phase and exhibits a significant increase at the site of implantation [68]. Unlike uNKs, macrophages can be detected in pregnancy. Macrophages play essential defensive functions, including chemotaxis, phagocytosis, secretion, and antigen presentation. They contribute to the elimination of endometrial microorganisms in the endometrium [69,70]. During pregnancy, macrophages in the decidua can regulate the killing effect of uNKs on trophoblast cells [71]. Both DCs and macrophages serve as antigen-presenting cells in the endometrium and play a significant role in inducing immune tolerance [72,73].

During pregnancy, the invasion of semi-allogeneic fetal extravillous trophoblasts (EVTs) into the uterus occurs without rejection via the maternal immune system. Initially, it was believed that this phenomenon was due to maternal immune tolerance towards cells expressing foreign fetal antigens [74]. However, subsequent studies have confirmed that pregnant mothers do develop cytotoxic T cells and antibody-mediated responses specific to fetal antigens [75]. These maternal immune responses to fetal antigens can be mitigated by inducing Tregs specific to fetal antigens and regulating effector T cells and NK cells at the maternal–fetal interface [76,77].

The immune cells have been found to express several receptors for HLA-G, including ILT-2, ILT-4, and KIR2DL4 (Table 2) [78]. Among the immune cells at the maternal–fetal interface, NK cells exhibit the highest expression of these three receptors. KIR2DL4 can be used as both an activation receptor and an inhibitory receptor. When HLA-G is membrane-bound, it interacts with KIR2DL4, resulting in the inhibition of decidual NK-cell-mediated cytolysis and the suppression of its cytotoxic effects [79]. It is worth noting that KIR exhibits a higher affinity for the recognition of HLA-C [80]. Since HLA-C is highly polymorphic, certain combinations of HLA-C with KIR may hinder trophoblast invasion, increasing the risk of various pregnancy disorders, including preeclampsia, fetal growth restriction, and recurrent miscarriage [81,82]. The study found that the nucleotide-binding domain leucine-rich repeat protein 2 (NLRP2) highly expressed on EVT can inhibit the cell surface expression of HLA-C without affecting the expression of HLA-E and HLA-G on EVT [83]. NLRP2 may establish immune tolerance at the maternal–fetal interface by reducing the immunosuppressive effects of NK and T cells. Additionally, dNK cells regulate trophoblast invasion by producing interleukin 8 and interferon-inducible protein 10 chemokines [84]. Recent studies have emphasized the significance of the dynamic balance of HLA-G on EVT and NK cells in ensuring immune tolerance at the maternal–fetal interface and facilitating virus immunity [85]. In addition to the direct effects of HLA-G binding to specific inhibitory receptors, HLA-G exerts an indirect immunosuppressive function via the expression of the non-classical HLA class I molecule, HLA-E. HLA-E directly binds to peptides derived from HLA-G, and the resulting HLA-E/peptide complex interacts with the inhibitory receptor CD94/NKG2A, which is predominantly expressed in NK cells [86].

**Figure 1:**
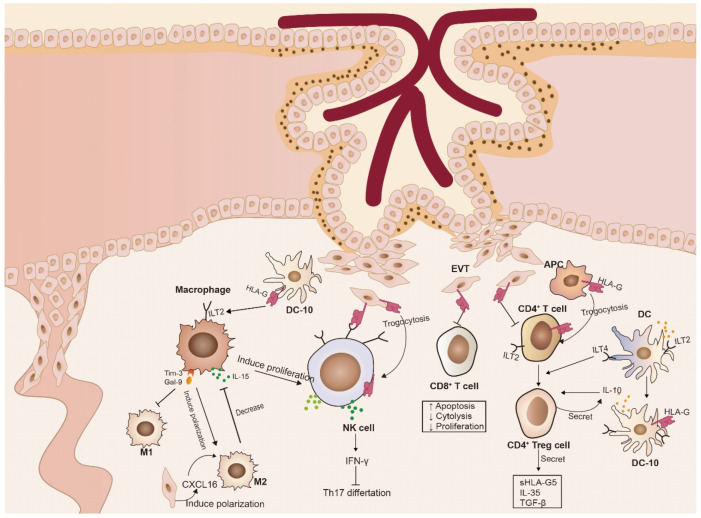
Schematic of the interaction between HLA-G and immune cells at the maternal–fetal interface. HLA-G promotes the differentiation of DCs into tolerogenic DCs (DC-10), secreting IL-10 and TGF-β and expressing more HLA-G. IL-10 and TGF-β, in turn, induce the generation of Tregs and inhibit the toxicity of NK cells. HLA-G inhibits T cell proliferation, induces apoptosis, and reduces cytolysis by binding to T cell ligands. Trogocytosis enables CD4^+^ and CD8^+^ T cells to acquire HLA-G1 from APCs. CD4^+^HLA-G^+^ Treg cells exert their inhibitory function by secreting various tolerant molecules such as sHLA-G5, IL-10, IL-35, and TGF -β. Decidual macrophages (dMΦs) produce IL-15, which induces NK cell proliferation. The CXCL16-mediated polarization of human M2 macrophages reduces the expression of IL-15, thus decreasing NK cell cytotoxicity. Tim-3 induces polarization of M2 cells, leading to an immune-tolerant microenvironment at the maternal–fetal interface and promoting trophoblast invasion. Following interaction with HLA-G, NK cells secrete senescence-related secretory phenotypes (SASP), which contribute to immune tolerance at the maternal–fetal interface, spiral artery remodeling, and fetal growth.

In addition to NK cells, several other immune cell types are involved in promoting tolerance at the maternal–fetal interface (Figure 1). HLA-G expressed at this interface can induce the apoptosis of T cells and promote the generation of suppressor T cells. Moreover, a significant accumulation of tolerant DCs occurs in the decidua. These DCs not only express HLA-G but also secrete IL-10, which plays a crucial role in inducing immune tolerance [95]. Macrophages, on the other hand, contribute to maternal–fetal immunity regulation by secreting various pro-inflammatory factors and interacting with T cells and NK cells.

### 3.2. HLA-G and dNK Cells

NK cells express two HLA-G receptors: KIR2DL4 and ILT2 [96]. Interestingly, despite the presence of these different receptors, HLA-G exerts similar effects, inhibiting the cytotoxicity of dNK cells and regulating spiral artery remodeling and fetal growth. HLA-G can attenuate the cytotoxicity of dNK cells independently of the KIR2DL4 and ILT2 receptors. The leader peptide of HLA-G exhibits a strong binding affinity for HLA-E, facilitating its promotion and stabilization on the cell membrane surface. HLA-E interacts with the inhibitory receptor NKG2A/CD94 on NK cells, thereby diminishing their cytotoxicity [97].

**Figure 2:**
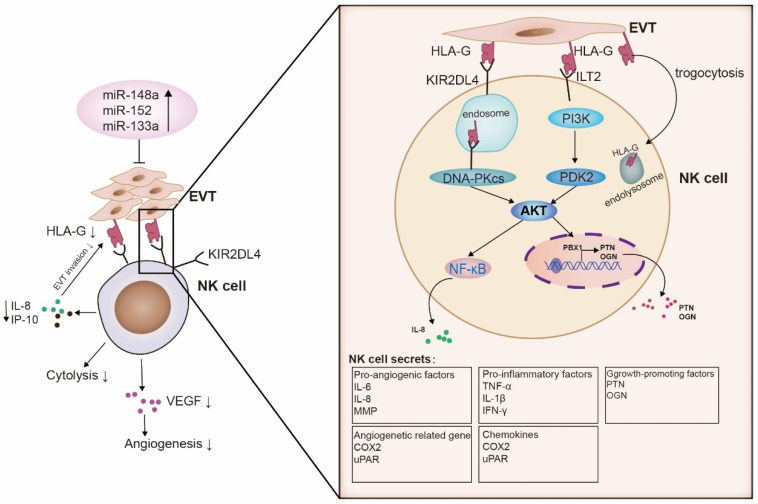
Schematic of the molecular interaction between HLA-G and decidual NK cells at the maternal–fetal interface. Several microRNAs (miR-133a, miR-152, and miR-148a) bind to the 3′ UTR of HLA-G, resulting in the downregulation of HLA-G expression in trophoblast cells. When miRNAs are bound to HLA-G, they influence the secretion ability of dNK cells upon interaction with KIR2DL4. KIR2DL4, in turn, interacts with DNA-PKcs and initiates a signaling pathway involving serine/threonine kinases DNA-PKcs and Akt, as well as NF-κB. This pathway leads to the expression of the senescence-associated secretory phenotype (SASP), which includes pro-inflammatory factors like TNF-α, IL-1β, and IFN-γ, as well as pro-angiogenic factors such as IL-6 and IL-8. In addition, ILT2 activates the PI3K-AKT signaling pathway, resulting in the secretion of the growth-promoting factors pleiotrophin (PTN) and osteoglycin (OGN), which facilitate early fetal growth. HLA-G-incorporated NK cells possess lower toxicity. NK cells that acquire HLA-G from EVT via trogocytosis have reduced cytotoxicity. They are important for maintaining the immune tolerance of the maternal–fetal interface and the viral immunity.

Numerous studies have extensively investigated the interaction between HLA-G and inhibitory receptors to suppress the cytotoxicity of maternal NK cells. The binding of HLA-G to KIR2DL4 plays a crucial role in inducing immune tolerance at the maternal–fetal interface. This interaction inhibits NK cell cytotoxicity, regulates cytokine secretion, controls trophoblast cell invasion, and maintains local immune suppression. A more detailed mechanism of how HLA-G and dNK cells interact has been suggested recently. It involves the regulation of pro-inflammatory and growth factors by microRNAs (miRNAs) that bind to HLA-G and senescence signals, thereby inhibiting the function of dNK cells and promoting fetal growth (Figure 2).

#### 3.2.1. MiRNAs

MiRNAs are a class of small non-coding RNAs that regulate gene expression via translational repression. The expression and functional impact of specific miRNAs on HLA-G and pregnancy-related diseases have been investigated. In the context of recurrent spontaneous abortion (RSA), miR-133a was found to be highly expressed in the villi of RSA cases. Using luciferase reporter gene analysis, miR-133a was shown to bind to the HLA-G 3′ UTR, resulting in the downregulation of HLA-G protein expression in human trophoblast cell lines (JEG-3 and HTR-8/SVneo). Co-culture experiments of dNK cells with miR-133a-transfected HTR-8/SVneo cells demonstrated reduced levels of IL-8, IP-10, and VEGF; the inhibition of HTR-8/SVneo cell migration; and decreased tube formation ability in human umbilical vein endothelial cells [98]. These findings suggest that miR-133a inhibits the function of dNK cells by downregulating HLA-G expression, potentially contributing to RSA development. Targeting miR-133a could be a promising therapeutic approach for RSA.

Compared with the normal placenta, miR-152 was upregulated in the preeclamptic placenta, and HLA-G was downregulated in the placenta. The overexpression of miR-152 resulted in increased NK cell-mediated cytolysis in JEG-3 cells [99]. This implies that miRNAs that bind to HLA-G with high expression may increase the risk of pregnancy-related diseases, such as RSA and pre-eclampsia (PE). These miRNAs could be potential targets for treating or preventing pregnancy-related diseases. Moreover, the level of miR-148a was significantly upregulated in both the placenta and peripheral blood of patients with intrahepatic cholestasis of pregnancy (ICP) [100]. Hence, using liposomes to deliver miR-148a and miR-152 into the uterus and downregulate HLA-G could be a novel contraceptive strategy [101].

#### 3.2.2. Growth-Promoting Factors

HLA-G has been shown to play a role in promoting fetal growth by stimulating the secretion of growth-promoting factors (GPFs) in NK cells, such as PTN and OGN. PTN is a heparin-binding protein that promotes angiogenesis in the microvasculature of the villous mesenchymal core [102]. In the amniotic fluid of chorioamnionitis, the expression level of PTN is low, suggesting that the abnormality of PTN may serve as a marker for intrauterine infection [103]. OGN, the small leucine-rich proteoglycan of the extracellular matrix, can modify cell behavior and regulate fibrosis by interacting with growth factors [104]. The transcription factor PBX1 was identified in decidual NK cells and was found to play a role in promoting the transcription of PTN and OGN in these cells, thereby further facilitating fetal development. Via screening using a signal pathway antibody chip, it was revealed that embryo-derived HLA-G signals could activate the PI3K-AKT signaling pathway of decidual NK cells via the ILT2 molecules present on the surface of these cells, leading to the upregulation of PBX1 expression [105]. The impairment of PBX1 in decidual NK cells has been found to be positively associated with the pathogenesis of unexplained recurrent spontaneous abortion (URSA) and may serve as a potential biomarker for this condition [105]. Further studies found that PBX1 recruited neutrophils to cause an inflammatory response by upregulating lipocalin 2 in dNK cells and then showed signs of fetal growth restriction [106].

HLA-G expressed by embryonic-derived EVTs interacts with decidual NK cells, inducing them to express various growth factors that contribute to embryonic development. This interaction between HLA-G and dNK cells further promotes the secretion of growth factors, supporting embryonic growth. During early pregnancy, both humans and mice exhibit significant populations of natural killer cells (CD49a^+^Eomes^+^NK cells) in the uterus [107]. These NK cells secrete abundant growth-promoting factors crucial for the early development of the fetus. When these growth factors are absent, it can lead to embryonic growth restriction or miscarriage. Fu et al. conducted a study demonstrating the essential role of decidual NK cells in producing growth factors that facilitate embryonic development [108]. Compared with normal pregnancy, RSA patients showed a decrease in the secretion of growth factors by dNK cells, which compromised their ability to support the normal development of early embryos. In vitro, decidual-like NK cells were induced with bone marrow hematopoietic stem cells, and intravenous adoptive transfusion was performed on mice. After the transfusion, both aged mice and growth-factor-deficient mice demonstrated significantly improved pregnancy outcomes, and embryonic growth restriction was relieved [108]. The human-induced NK cells displayed high expression of growth-promoting factors and pro-angiogenic factors in the mouse pregnancy model, leading to enhanced fetal growth and improved uterine artery blood flow [109]. These findings highlight the critical role of decidua NK cells in supporting embryonic development and could be a potential treatment for patients experiencing adverse pregnancy outcomes.

#### 3.2.3. Senescence Signal

The senescence signal is another important signal activated by the interaction of HLA-G and the receptor. Senescent cells are metabolically active and secrete a series of soluble mediators called the senescence-associated secretory phenotype (SASP) [110]. The SASP includes various cytokines, such as pro-inflammatory cytokines, growth factors, chemokines, and matrix-remodeling enzymes [111]. The functions of the senescence signal generated by the interaction of HLA-G and KIR2DL4 receptors at the maternal–fetal interface are as follows: (1) During implantation, fetal trophoblast cells produce soluble HLA-G (sHLA-G). The KIR2DL4 receptor on the surface of NK cells endocytoses sHLA-G into small vesicles, activating NK cells and triggering a DNA damage response (DDR) signaling pathway. The DDR signaling induces the expression of p21 and phosphorylation of HP1-γ, resulting in NK cell senescence. Senescent NK cells secrete important factors that promote and enhance the senescence state, such as IL-6, IL-8, IL-1β, and p21. These senescence signals can promote vascular remodeling by increasing endothelial vascular permeability [112,113]. (2) This activation leads to the production of SASP components by NK cells, including pro-inflammatory factors such as TNF-α, IL-1β, and IFN-γ, as well as pro-angiogenic factors like IL-6 and IL-8 [91]. (3) The interaction between HLA-G and the KIR2DL4 receptor also activates proteins of the urokinase plasminogen activator system, such as uPAR and COX-2 [114]. uPAR facilitates the breakdown of the extracellular matrix required for trophoblast cell invasion and spiral artery remodeling. COX-2, on the other hand, is the rate-limiting enzyme in prostaglandin biosynthesis, and NF-κB stimulates its expression in human endometrial stromal cells (ESCs). COX-2 is essential for angiogenesis in mouse decidualization [115,116]. Therefore, the SASP promoted fetal angiogenesis, and senescence contributed to a successful pregnancy in this process.

Senescent dermal fibroblasts express the non-classical MHC molecule HLA-E, which interacts with the inhibitory receptor NKG2A expressed by NK cells to inhibit the immune response to senescent cells. In vitro, blocking the interaction between HLA-E and NKG2A can enhance the immune response to senescent cells [117]. The effect of blocking the KIR2DL4-sHLA-G interaction at the maternal–fetal interface on the senescent signal is currently unknown. Senescent cells can be utilized by the immune system to efficiently inhibit DCs or other immune cells in the decidual stromal cells of the uterus to promote embryo implantation. However, when stromal cells become senescent, they may acquire APC-like activity, allowing senescent cells to be cleared more precisely from the pregnant endometrium and preserving the viability of the developing embryo [118,119]. Therefore, the SASP produced by the KIR2DL4-sHLA-G interaction could contribute to normal vascular adaptation to support fetal development during pregnancy.

Senescence-deficient mice display morphological aberrations in placental labyrinths, and the downregulation of the senescence pathway has been observed in the human placenta of pregnancies affected by intrauterine growth restriction [120]. These findings suggest that the molecular pathway of aging regulates the structure and function of the placenta. During the rapid growth of the proliferative phase of the endometrium, endometrial stromal cells (ESCs) experience replication stress, leading to cellular senescence and the secretion of the senescence-associated secretory phenotype (SASP). IL-15 activates uNKs to selectively target and eliminate senescent decidual cells via granule exocytosis, facilitating the removal of senescent decidual cells and the remodeling of the endometrium during embryo implantation, thus ensuring normal embryonic development [119].

Recent studies utilizing in vitro models and patient cell lines from in vitro fertilization (IVF) have revealed that the presence of senescent ESCs in the endometrium and the secretion of the SASP impair the hormone responsiveness of the tissue and interfere with embryo implantation. uNK cells can recognize and eliminate pro-inflammatory senescent decidual cells via the CD44 receptor, thus promoting successful implantation [121]. The application of drugs (senomorphics) that inhibit senescence-related secretory phenotypes during the proliferative phase of the menstrual cycle may offer a potential strategy for enhancing successful embryo implantation [122]. It is worth noting that evaluating the senescence index of decidual NK cells at different stages of embryonic development could provide further insights into the role of senescence in placental development.

#### 3.2.4. Trogocytosis

HLA-G plays a crucial role in regulating immune tolerance at the maternal–fetal interface to support fetal development. In addition to its immune regulatory function, HLA-G is involved in a unique cellular process called trogocytosis, which involves the rapid uptake of membrane-bound molecules upon direct contact. Activated NK cells can acquire HLA-G1 from tumor cells via trogocytosis, resulting in the cessation of proliferation, loss of cytotoxicity, and acquisition of inhibitory effects on other NK cells [123]. This functional alteration in NK cells is mediated by the specific binding of HLA-G1 to ILT2 receptors on their surface, leading to immunosuppressive effects. The phenomenon of trogocytosis also exists in the maternal–fetal interface. Studies have indicated that HLA-G plays a role in balancing immune tolerance and antiviral immunity at the maternal–fetal interface [85]. Notably, apparent contacts between filopodia projections from EVT and dNK cells have been observed in co-culture experiments. HLA-G protein is detected on the surface and inside dNK cells, while HLA-G mRNA is not detected, suggesting that dNK cells acquire HLA-G from EVTs via trogocytosis. The transfer of HLA-G from EVTs to dNKs in co-culture can be activated by adding IL-15. However, after 36 h, the surface and intracellular HLA-G in dNKs was lost or degraded, and cytotoxicity was recovered. These results suggested that during placental virus infection, the cytolytic ability of dNKs can be restored by activating pro-inflammatory cytokines such as IL-15 [85].

The interaction between HLA-G and immune cells allows the fetus to adapt to the maternal environment better. When immune tolerance towards the fetus is compromised, it can negatively impact embryonic growth and development and even result in miscarriage. Elevated levels of sHLA-G can be detected in the plasma of pregnant women, and the levels of sHLA-G detected in women with RSA are lower than those in women with only one abortion. Particularly, sHLA-G1 is not present in the plasma of women with RSA [124]. However, patients with a history of recurrent miscarriage exhibit increased levels of HLA-G protein expression in the full-term placenta after successful delivery [125]. These findings suggest a direct association between HLA-G and abortion. The decrease in sHLA-G levels may disrupt the balance between pro-inflammatory and anti-inflammatory factors at the maternal–fetal interface, leading to immune disorders and triggering miscarriage.

### 3.3. HLA-G and T Cells

Decidual T cells account for 5–15% of placental white blood cells in the first trimester of pregnancy, and this number can reach 70% during term pregnancy [126]. T cells can be categorized into CD4^+^ T cells and cytotoxic CD8^+^ T lymphocytes. Among CD4^+^ T cells, CD4^+^ Treg cells act as immunosuppressive cells, and they accumulate in the placenta of the uterus during pregnancy to prevent fetal immune rejection and maintain a normal pregnancy [127,128]. Research has identified three distinct types of decidual Tregs (CD25^HI^FOXP3+, PD1^HI^IL-10+, and TIGIT^+^FOXP3^dim^) that suppress effector T cell responses in human pregnancy. The co-culture of HLA-G+EVT directly increased the ratio of CD25^HI^FOXP3+ Tregs compared with T cells cultured separately [66]. CD25^HI^FOXP3+ Tregs play a role in suppressing both fetal-specific and nonspecific immune reactions during pregnancy [129]. HLA-G has the ability to inhibit T lymphocyte cytotoxicity and the proliferation of allogeneic CD4^+^ T cells [130,131]. Significantly, the receptor ILT2, which exhibits a high affinity for HLA-G, is expressed on the surface of certain CD4^+^ and CD8^+^ T cells [132,133].

CD4^+^ and CD8^+^ T cells have the ability to acquire immunosuppressive HLA-G1 from APCs via trogocytosis within a short time span, enabling T cells to reverse their function from effectors to regulatory cells [134]. These T cells have low proliferation and exert potent immunosuppressive effects within inflammatory sites, indicating their crucial role in regulating local inflammatory responses in vivo [42]. During a successful pregnancy, there is notable accumulation of CD4^+^ HLA-G^+^ T cells in the maternal decidua, contributing to the establishment of fetal immune tolerance [95]. An analysis of peripheral blood mononuclear cells in pregnant women with PE has revealed decreased levels of HLA-G and reduced frequencies of regulatory CD8^+^CD28^−^T cells. These observations may be associated with the elevated levels of pro-inflammatory cytokines (IL-2, IL-4, IL-6, IL-10, IFN-γ, TNF-α, and IL-17) observed in PE [135]. The dysregulation of cytokines in PE can disrupt immune homeostasis and contribute to immune dysregulation.

Tregs derived from the maternal–fetal interface exhibit a distinct differentiation phenotype reminiscent of tumor-infiltrating Tregs (TITRs). A comparative analysis of Treg transcriptional profiles in the placenta and those of TITRs has revealed a significant overlap between these two populations. TITRs are currently the focus of extensive research as targets for tumor immunotherapy [136]. By leveraging insights from tumor research, a deeper understanding of pregnancy-related immune disorders can potentially be gained.

### 3.4. HLA-G and DCs

Decidua in the placenta also contains DCs, which play a crucial role in maintaining and regulating maternal–fetal tolerance. DCs achieve this by inducing T cell dysfunction and apoptosis, as well as promoting the generation of Tregs.

HLA-G binds to ILT2 and ILT4 on DCs, and the interaction with ILT4 inhibits DC maturation while facilitating the differentiation of CD4^+^ T cells into Tregs [137]. Du et al. discovered a feedback loop between multiple immune cells mediated by DCs, inducing a tolerance immune response and maintaining the immune balance between the fetus and mother [138]. Human trophoblast cells express thymus stromal lymphopoietin (TSLP), which stimulates DCs to promote Treg differentiation in early pregnancy. Treg can inhibit the proliferation of CD4^+^CD25^−^ T cells, suppress IFN-γ secretion, and induce trophoblast cells to express HLA-G. This preferentially promotes the production of Th2 cytokines and reduces the cytotoxicity of decidual CD16^−^CD56^bright^ NK cells.

Gori et al. demonstrated the presence of a distinct subset of tolerogenic DCs that accumulate in human decidua during pregnancy [72]. These specialized cells, referred to as DC-10 cells, express HLA-G and secrete significant amounts of IL-10. In the maternal decidua during the first trimester of pregnancy, there is a higher abundance of DC-10 cells, which play a crucial role in regulating Tregs [95]. Tr1 cells are a type of Treg cell that differentiate upon antigen stimulation and are induced by IL-10. They are characterized by their ability to produce high levels of IL-10 and TGF-β. DC-10 cells express both ILT4 and HLA-G and possess the unique capacity to induce Tr1 cell differentiation, thereby playing a pivotal role in maintaining tolerance to self and non-self antigens [137].

Decidual DC-10 cells play an important role in maintaining maternal–fetal immune tolerance [139,140,141]. They exert the effects on NK cells in the decidua via HLA-G, leading to the inhibition of NK cell cytotoxicity and cytokine expression [142,143]. Furthermore, the secretion of pro-angiogenic factors by DC-10 cells contributes to fetal growth [144]. DC-10 cells interact with dNKs or dMΦs via ILT2–HLA-G, promoting their activation and the release of angiogenic factors. A study has indicated that CD14^+^DC^-^SIGN^+^APC cells in decidual tissue exhibit phenotypic similarities to DC-10 cells and express high levels of HLA-G and ILT4. However, further investigation is required to determine whether they belong to the same cell population [145]. The percentage of DC-10 cells in the decidua is significantly higher than that in peripheral blood [72,141]. Additionally, CD4^+^T cells obtain HLA-G molecules from DC-10 via the trogocytosis, resulting in the generation of CD4^+^HLA-G^+^T cells, which play a role in inducing immune tolerance between the mother and fetus and maintaining a healthy pregnancy. However, this phenomenon is not observed in pregnant women with PE [146].

### 3.5. HLA-G and Macrophages

Decidual macrophages (dMΦs) constitute ~20% of decidual leukocytes [147]. They play important roles in immune suppression, tissue repair, the secretion of various factors, and the promotion of placental growth and trophoblast cell invasion. Unlike NK cells, macrophages do not express KIR2DL4 on their surface; however, they can interact with HLA-G via ILT2, leading to the production of pro-inflammatory cytokines [148,149,150,151]. dMΦs contribute to maintaining a healthy pregnancy via specific functions such as inducing tolerance, remodeling spiral arteries, and promoting embryo implantation. sHLA-G5 promotes macrophage activation, leading to the downregulation of M1 macrophage markers and an increase in M2 macrophage markers. Macrophages polarized by sHLA-G5 exhibit enhanced phagocytic activity, which plays a significant role in regulating maternal–fetal tolerance and placental development [152]. Moreover, pregnancy-related hormones induce the polarization of decidual macrophages into M1 or M2 phenotypes. dMΦs can produce IL-15, which stimulates the proliferation of NK cells. In early pregnancy, trophoblast-derived CXCL16 induces the polarization of human M2 macrophages. Polarized M2 macrophages downregulate IL-15, leading to the inactivation of NK cells and a reduction in their cytotoxicity [153].

T cell immunoglobulin mucin domain 3 (Tim-3) serves as a negative regulator of costimulatory signaling molecules. It is expressed on various immune cells at the maternal–fetal interface and plays a role in macrophage polarization regulation [154]. Li et al. conducted a study showing that Tim-3 is capable of inducing M2 macrophage polarization, creating an immune-tolerant microenvironment at the maternal–fetal interface, promoting trophoblast invasion, and facilitating uterine spiral artery reconstruction to ensure successful pregnancy. In mice with PE, a condition characterized by elevated blood pressure and organ damage during pregnancy, Tim-3 expression was found to be decreased in dMΦs. Deficiency in the Gal-9/Tim-3 signaling pathway resulted in macrophage polarization towards the M1 phenotype, the disruption of immune tolerance at the maternal–fetal interface, compromised trophoblast invasion, and impaired spiral artery reconstruction, ultimately leading to the development of PE. However, when Gal-9 was administered exogenously, it activated the Tim-3 signaling pathway, reversed the polarization of dMΦs towards M2 phenotype, induced maternal–fetal immune tolerance, and prevented the onset of PE [155].

## 4. Conclusions

In this review, we provide a systematic overview of the regulatory mechanisms of HLA-G on NK cells during early pregnancy: (1) MicroRNAs downregulate HLA-G to inhibit decidual NK cell function. (2) HLA-G stimulates the secretion of growth-promoting factors to maintain fetal growth. (3) HLA-G interacts with NK cell receptors to generate senescence signals and promote fetal angiogenesis. (4) NK cells acquire HLA-G from EVTs via endocytosis, thereby promoting immune tolerance and antiviral immunity at the maternal–fetal interface. In addition, we explore the mechanism by which HLA-G regulates the function of T cells, dendritic cells, and macrophages. Furthermore, a process in which different types of immune cells interact by secreting cytokines to establish a tolerance loop and promote an immune tolerant environment during pregnancy has been described. There is a complicated two-way regulatory mechanism between HLA-G and IL-10. HLA-G-modified DCs induce IL-10-dependent Treg cells and, in turn, stimulate the generation of tolerant DCs, thus forming a loop of immune tolerance. The specific molecular mechanism of the interaction between tolerant DCs and Treg cells in this loop is still unclear, and the regulatory mechanism of this amplification effect of the tolerance loop requires further study.

In 2019, Niu et al. conducted a meta-analysis to investigate whether the presence of sHLA-G in the embryo culture medium could predict the clinical outcome of IVF [156]. The findings indicated that the presence of sHLA-G in the embryo culture medium was associated with higher implantation and pregnancy rates. Moreover, the placenta releases extracellular vesicles that facilitate communication between the embryo and the mother. HLA-G has been found to be expressed in extracellular vesicles derived from human cytotrophoblast cells. These extracellular vesicles can enhance the release of inflammatory factors from decidual stromal cells, which may play a significant role in maintaining pregnancy [157]. However, it is important to note that further clinical studies are necessary to validate and confirm these conclusions. Additionally, more accurate methods for detecting sHLA-G should be employed, and there is a need for further research on the molecular mechanisms of sHLA-G in early embryonic development and implantation.

NK cells are abundantly expressed at the maternal–fetal interface and promote successful reproduction via a series of receptor ligands. Therefore, in future studies, the regulation of NK cell function by HLA-G will be explored as a breakthrough to further reveal the interaction between NK cells and other decidual immune cells. A deeper elucidation of the molecular mechanism by which immune cells at the maternal–fetal interface induce immune tolerance and maintain pregnancy will help explain the exact role and significance of HLA-G in pregnancy and pregnancy-related diseases. A variety of immune cells have receptors that bind to HLA-G (especially KIR2DL4), which can regulate the activity of immune cells in pregnancy and pregnancy-related diseases. Consequently, HLA-G holds promise as both a therapeutic target and a clinical indicator for pregnancy and pregnancy-related diseases; however, further research is required to translate these findings into practical applications.

## Figures and Tables

**Table 1:** HLA-G isoforms and detected expression.

HLA-G Isoforms	Types	Protein Isoforms	Expressed Cells
HLA-G1	Membrane-bound	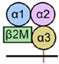	Trophoblasts [27] Tumor cell lines [28] Antigen-presenting cells (APC) [29]
HLA-G2 HLA-G3 HLA-G4	Membrane-bound	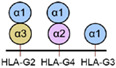	Cytotrophoblast [30] Tumor cell lines [31]
HLA-G5	Soluble	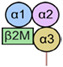	Body fluids (such as amniotic fluid and serum) [32] Trophoblasts [33] Thymus [10] Oocytes [34] Pre-implantation embryo [35]
HLA-G6	Soluble	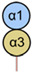	Maternal blood during pregnancy [36] Serum from heart-transplanted patients [37]
HLA-G7	Soluble	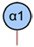	Transfected cell supernatant [38] Maternal blood during pregnancy [39]

**Table 2:** Signaling cascades following HLA-G interaction with its receptors of immune cells.

HLA-G Receptors	Immune Cells	Signal Pathway	Function
ILT-2	NK cell	Cytoskeleton rearrangement	Inhibition of NK cytolysis [87]
	T cell	ILT-2/ILT-4/FasL	Inhibition of T-cell alloproliferation [88]
	B cell	SHP-1/AKT, mTOR, c-Raf, GSK3β, and Foxo pathways	Regulating B cell fate decision, such as cell proliferation and differentiation [89]
ILT-4	T cell	IL4/SHP-1/2-IL-6--STAT3	Modulation of dendritic cell differentiation [90]
KIR2DL4	NK cell	(DNA-PKcs)-Akt-NF-κB	Pro-inflammatory response or proangiogenesis [91]
		Blocking MAPK and DNA-PKcs	Inhibition of killing activity of NK and promotion of inflammatory cytokines secretion [91]
		Syk/MAPK/ERK	Inhibition of NK cytotoxicity [92]
Uncertain		Fas/FasL	Apoptosis of NK cells [93]
		SHP-2/mTOR	Inhibition of lymphocyte cell cycle [94]

## Data Availability

Not applicable.
